# Radioactivity distribution and concomitant hazards evaluation of industrial zones soils from Chattogram, Bangladesh: A multivariate statistical analysis

**DOI:** 10.1371/journal.pone.0328356

**Published:** 2025-07-16

**Authors:** M.M. Mahfuz Siraz, Shahidul Islam, Afroza Shelley, Mohammad Shafiqul Alam, Araf Mahmud, Md. Bazlar Rashid, Mayeen Uddin Khandaker, Selina Yeasmin, M. Safiur Rahman

**Affiliations:** 1 Health Physics Division, Atomic Energy Centre Dhaka, Dhaka, Bangladesh; 2 Department of Nuclear Engineering, University of Dhaka, Dhaka, Bangladesh; 3 Department of Nuclear Engineering, Chittagong University of Engineering & Technology, Chattogram, Bangladesh; 4 Department of Civil Engineering, Dhaka International University, Dhaka, Bangladesh; 5 Geological Survey of Bangladesh, Segunbaghicha, Dhaka, Bangladesh; 6 Faculty of Graduate Studies, Daffodil International University, Daffodil Smart City, Birulia, Savar, Dhaka, Bangladesh; 7 Applied Physics and Radiation Technologies Group, CCDCU, Faculty of Engineering and Technology, Sunway University, Bandar Sunway, Selangor, Malaysia; 8 Department of Physics, College of Science, Korea University, 145 Anam-ro, Seongbuk-gu, Seoul, Republic of Korea; 9 Water Quality Research Laboratory, Chemistry Division, Atomic Energy Centre Dhaka, Dhaka, Bangladesh; 10 Faculty of Engineering, Daffodil International University, Daffodil Smart City, Birulia, Savar, Dhaka, Bangladesh; Ural Federal University named after the first President of Russia B N Yeltsin Institute of Physics and Technology: Ural'skij federal'nyj universitet imeni pervogo Prezidenta Rossii B N El'cina Fiziko-tehnologiceskij institut, RUSSIAN FEDERATION

## Abstract

Soil can pose significant radiation hazard in areas with elevated radioactivity levels from geological or anthropogenic sources, potentially contributing to human exposure through the food chain and atmosphere. However, industrial activities can alter radionuclides distribution by releasing residues or effluents, leading to their accumulation in the environment. In general, soil provides clear insights into geological characteristics and heavy metal exploration, in addition to assessing the risks of radiation exposure. This study investigates the distribution of NORMs and assesses radiological hazards in twenty soil samples collected from two major industrial zones in the Chattogram City of Bangladesh: the Bayazid Industrial Area and the Kalurghat Heavy Industry Area. The activity concentrations of ^226^Ra, ^232^Th, and ^40^K in the analyzed soil samples range from 8 ± 1–18 ± 1, 15 ± 1–35 ± 3, and 192 ± 17–420 ± 35 Bq/kg, respectively, remaining below the global average for soil. The radiological hazard indices indicate negligible health risks to the public or environment, suggesting that the industrial activities are not releasing any radiotoxic elements in the surrounding environment. Statistical analysis identified ^40^K and ^232^Th as the primary contributors to radiological hazards, supported by strong correlations and significant principal component loadings. Additionally, this study provides baseline data for monitoring environmental radioactivity levels, particularly in light of the upcoming commissioning of the Rooppur Nuclear Power Plant in 2025.

## 1. Introduction

Natural radioactivity is characterized by the spontaneous decay of unstable atomic nuclei and is a ubiquitous environmental phenomenon [1]. Natural radiation exposure to humans is widespread and diverse, resulting from a range of notable sources, including terrestrial (such as building materials, water, air and foodstuffs), extra-terrestrial (e.g., cosmic radiation), and internal radiation (such as ^40^K) [2]. The main external source of human irradiation is terrestrial background radiation, which is mostly sourced from naturally occurring radioactive materials (NORM) such as ^40^K and the decay products of ^238^U and ^232^Th series. Nearly 85% of the annual radiation dose that humans receive comes from natural radionuclides, which include both terrestrial and cosmogenic sources. Overall, natural radiation sources comprise about 80% of total human radiation exposure, with artificial sources accounting for the remaining 20% [3].

Radiation exposure has significant and dose-dependent biological effects. While both acute and chronic exposures have been epidemiologically associated to a variety of malignancies, including different kinds of leukemia and organ-specific tumors, as well as those affecting the thyroid, breasts, and lungs, high levels have the potential to cause cellular death [4]. Research has shown that higher radiation exposure levels are linked to a higher risk of developing cancer, suggesting that exposure over the average of natural background radiation worldwide is linked to a higher chance of developing cancer [4,5]. This connection emphasizes how crucial it is to track and control radiation exposures, both natural and man-made, in order to reduce any possible health hazards. Natural radiation makes up the majority of human exposure patterns, which emphasizes how important it is to comprehend and measure background radiation levels for both public health evaluation and efficient radiation protection.

Depending upon the variation in the geology, the NORM activity concentration in earth crust can greatly show a discrepancy from region to region [6–8]. The primary sources of natural radioactivity in earth crust are ^226^Ra, ^232^Th, and ^40^K. Due to the non-uniformity of the distribution of these radionuclides in the environs, it is important to measure and analyze their activity concentrations in various locations. While ^226^Ra, ^232^Th, and ^40^K are major natural contributors, total radiation inventory also includes cosmic rays from space, radon gas in the atmosphere, and human-made sources [9,10]. These artificial sources include nuclear power plants, medical procedures, and industrial activities. The industrial sector is particularly important, as in most manufacturing processes, radioactive materials could be generated, or the existing ones could be concentrated during operations [1], leading to products and waste materials that have higher levels of radioactivity than the original raw materials [11]. These concentrated materials are known as technologically enhanced naturally occurring radioactive materials (TENORM). The term “technologically enhanced” is used to highlight that these naturally occurring radionuclides have been concentrated through industrial activities. When industrial waste is dumped into nearby low-lying areas without adequate treatment, it can significantly contaminate the soil by increasing levels of TENORM [5]. This untreated waste presents serious risks, damaging the ecosystem’s macrophytes and soil fauna, while also posing potential health threats to human populations. Several major industrial sectors have been identified as primary sources of TENORM [6]. Industries such as fossil fuel energy production, phosphate processing, textile manufacturing, fabric and knit production, footwear manufacturing, medical disposable products, and oil and gas extraction, all contribute to the release of radioactive waste into the environment [12]. This situation necessitates stringent environmental management protocols to mitigate potential risks to ecosystems and human health. With rising public awareness and concern regarding environmental quality, it has become increasingly crucial to assess the impacts of radioactive waste discharge, even when it involves NORMs. Consequently, thorough monitoring and management strategies are vital to ensure both environmental and public safety.

A survey of literature shows that a thorough investigations into natural radionuclides in industrial soil matrices have been carried out in various geographical locations, including studies in Bangladesh [5,13–18] and other regions worldwide [19–29]. A study carried out in China has reported higher levels of ^232^Th and ^40^K in industrial areas [30]. Similarly, research in Bangladesh has shown increased concentrations of ^226^Ra, ^232^Th, and ^40^K in soil samples near industrial sites [5,17,18]. Investigations carried out in Spain [28], Russia [29], and Saudi Arabia [31] have revealed the presence of ^137^Cs contamination in soils near industrial areas, highlighting the uneven distribution of radionuclides linked to industrial activities across various regions. A prior study in Chattogram [32] noted the presence of ^137^Cs along with increased levels of ^232^Th in soil samples. Despite these important findings, there is still a considerable gap in our understanding of how NORMs are distributed within the industrial zones of Chattogram.

Chattogram, Bangladesh’s principal port city, encompasses strategically positioned industrial zones in Kalurghat, Bayezid, and Nasirabad. This comprehensive radiological assessment establishes a crucial baseline through precise documentation of current NORM concentrations in Chattogram, thereby facilitating the detection of temporal variations in radiation levels, particularly within industrially impacted zones. In this regard, our investigation focused on determining the distribution of NORMs in soil matrices proximate to Chattogram’s industrial zones. The findings may contribute to the development of targeted mitigation strategies aimed at reducing radiological hazards to ensure the protection of local populations and natural resources. Further, this research work may contribute to and be an excellent reference for radiological assessments at other important sites, like the Rooppur Nuclear Power Plant, and for the betterment of overall knowledge regarding radiological risk management and environmental sustainability related to industrial areas.

## 2. Methodology

### 2.1 Study area

**Fig 1:**
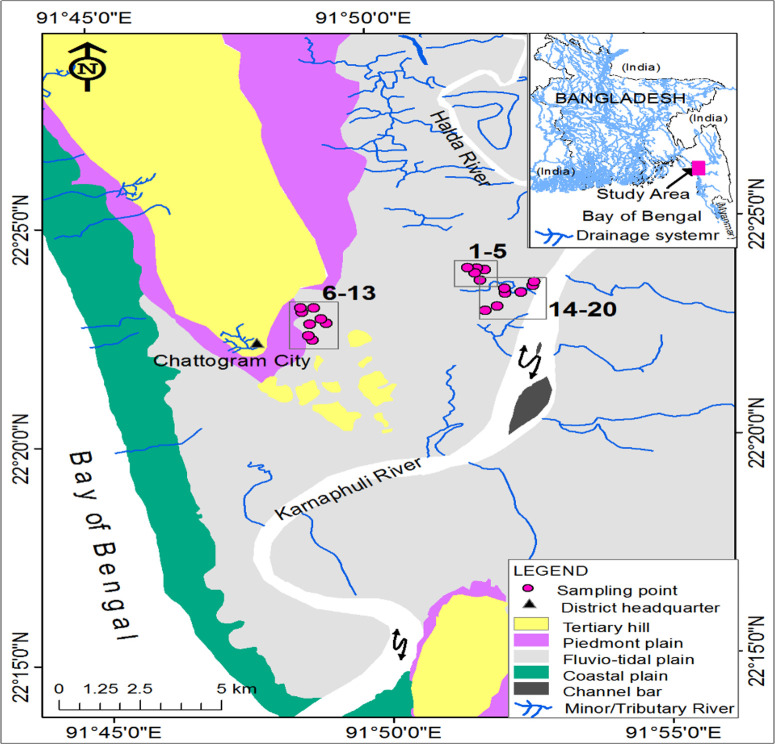
Geomorphology of the study area and its surrounding, and sampling points (modified after Siddique et al., 2021 [33]).

The present research area (Fig 1) is situated in the Folded Flank Tectonic Element of Bengal Basin [34]. The area is composed of recent coastal plain sediments along the west of the eastern coast (Cliff coast) backed by the hilly terrain which is composed of Miocene to Plio-Pleistocene sediments. Piedmont plain deposits are present in front of hilly terrains. Coastal plain sediments are enriched of silty clay and slight sand mixed with piedmont sediments, whereas the hilly terrain consists of inter-layered shale, sandstone, claystone, siltstone, silty shale, etc. The major portion of the area consists of recent fluvio-tidal plain and the sediments of the plain are mainly silty clay and clayey silt. Rashid et al. (2023) [35] noted that the recent sediments were deposited under tidal conditions and fluvial influences. These sediments originate from felsic-dominated metamorphic rock sources associated with the tectonic environment of continental island arcs, active continental margins, and oceanic islands. The sediments are categorized as shale, Fe-rich shale, and wake, respectively.

### 2.2 Sampling and preparation procedure

Twenty soil sampling was conducted across two distinct industrial zones: Kalurghat and Bayazid. The soil sampling procedure involved delineating a 1.0 m × 1.0 m quadrat at each sampling point, followed by surface clearing. Multiple soil samples were collected randomly within each quadrat at a depth of 05–10 cm and thoroughly homogenized to ensure representative sampling. To minimize analytical interference, the samples underwent preliminary processing to remove extraneous materials including stones, plant materials, glass fragments, organic debris, and lithic components. Each processed sample was subsequently stored in labeled polyethylene bags to prevent cross-contamination. Sample labels documented essential metadata including geographical location, unique sample identifier, and collection date. The samples were subsequently sent to the Health Physics Division of the Atomic Energy Centre Dhaka (AECD) for further processing. Initially, the samples were sun-dried naturally, followed by oven-drying at 105°C to 110°C at AECD. The samples were dried to a constant mass, pulverized, and standardized using a 2-mm mesh sieve. Approximately 500 g of each processed sample was placed in a plastic beaker, which was then sealed with PVC tape to prevent the escape of the gaseous radioisotopes [36]. The sealed samples were stored for 40 days to ensure secular equilibrium between ^222^Rn and its short-lived daughter nuclei prior to gamma spectroscopic analysis [37,38].

### 2.3 Measurement procedures and data analysis

**Fig 2:**
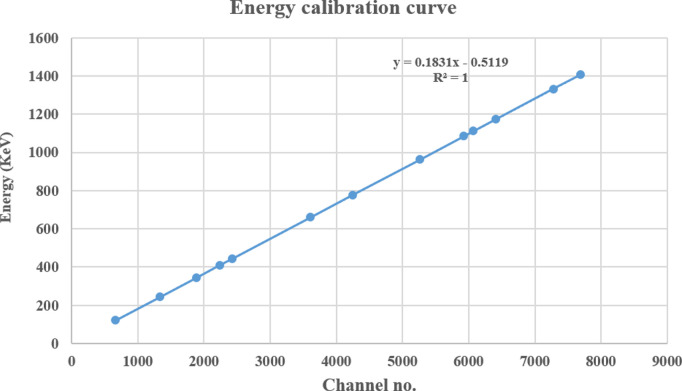
Energy calibration curve of the HPGe detector.

**Fig 3:**
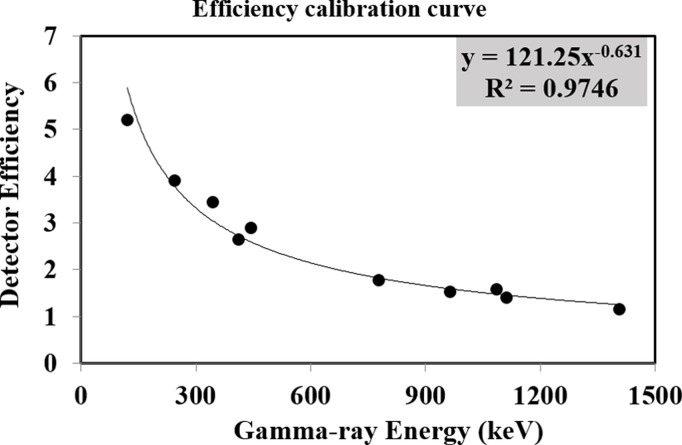
Efficiency calibration curve of the HPGe detector.

A high-resolution HPGe detector was used to measure the amount of gamma-ray-emitting radionuclides present in the samples. To shield the detector from outside radiation, a Pb tube-shaped structure was placed over it. This construction had a fixed bottom and a sliding cover at the top. To calibrate the detector’s energy response, point sources of ^22^Na, ^133^Ba, ^57^Co, ^60^Co, ^137^Cs, and ^152^Eu, each with an activity of 1 microcurie, were used. The detector’s efficiency for soil samples was evaluated using a known activity of ^152^Eu in an Al_2_O_3_ matrix, prepared in containers identical to those used for the actual samples [39]. Additional information regarding the fitting process and efficiency calibration has been detailed elsewhere [40]. The energy and efficiency calibration curves of the HPGe detector are presented in Figs 2 and 3.

The following equation (1) has been used to determine the activity concentration for each radionuclide [41–46]:


$$A_{i}=\;\frac{N^{\prime }}{\varepsilon \times \rho_{\gamma }\times w}$$ (1)

Here *A*_*i*_ (Bqkg^-1^) = the specific activity, $N^{\prime }$= the net count rate per second (sample – background), ε = HPGe detector’s efficiency, *ρ*_*γ*_ = γ-ray emission probability, and *w* = mass of the sample (kg).

The mathematical expression for estimating the uncertainty of the determined radioactivity, as described in [47,48] is provided in Eq. (2).


$$\begin{array}{c} Uncertainty\;=A_{i}\times \;\sqrt{{\left(\frac{u(N)}{N}\right)}^{2}+{\left(\frac{u(T)}{T}\right)}^{2}+{\left(\frac{u\left(\rho_{\gamma }\right)}{\rho_{\gamma }}\right)}^{2}+{\left(\frac{u(w)}{w}\right)}^{2}+{\left(\frac{u(\varepsilon )}{\varepsilon }\right)}^{2}} \end{array}$$ (2)

The sample counts, sample mass, counting time, γ-ray ray emission probability, and counting efficiency are represented by the symbols N, w, T, ρ_γ_, and ε, respectively.

### 2.4 Radiological hazard parameters

#### 2.4.1 Radium equivalent activity (Ra_eq_).

The expression of the overall radioactivity of the soil samples with respect to the hazardous radium is called radium equivalent activity. Equation (3) has been used to determine the radium equivalent activity in the samples [49,50].


$${Ra}_{eq}=\;S_{Ra}+1.43S_{Th}+\;0.077S_{K}$$ (3)

The mean activities of ^226^Ra, ^232^Th, and ^40^K are represented by *S*_*Ra*_, *S*_*Th*_, and *S*_*K*_ in Bq/kg, respectively.

#### 2.4.2 The absorbed dose rate.

It is assumed that human being is always receiving exposure to gamma radiation emitted from the radionuclides available in the soil. It means the presence of radionuclides in the soil possesses a relationship with the radiation exposure and possible health hazards. This relation has been characterized by a quantity called outdoor absorbed dose rate (D_out_). The outdoor absorbed dose rate from gamma-ray exposure at one meter above the ground was calculated using Equation (4) [51,52]:


$$D_{out}=\;{0.427S}_{Ra}+\;0.662S_{Th}+\;0.0432S_{K}$$ (4)

By using the D_out_ value, it is also possible to calculate indoor exposure, $D_{in}$ [44].


$$D_{in}=1.4D_{out}$$ (5)

#### 2.4.3 The annual effective dose.

The measured exposures outside and indoors, respectively, can be used to compute the annual effective doses. Therefore, using Eqs. (6) and (7), the yearly effective doses E_in_ (mSv/y) and E_out_ (mSv/y) were calculated [3,53].


$$E_{in}\left(\frac{mSv}{y}\right)=\;D_{in}\times (\;8760\;\times 0.7\;\times 0.8)\times 10^{-6}$$ (6)

$$E_{out}\left(\frac{mSv}{y}\right)=\;D_{out}\times (\;8760\times 0.7\;\times 0.2)\times 10^{-6}$$ (7)

Globally, the average annual effective dose of all terrestrial radiation, both indoors and outdoors, is 0.48 mSv/y [3]. In situations involving public exposure, various organizations advocate a yearly effective dose limit of 1 mSv/y [3,54–60].

#### 2.4.4 Hazard indices.

Assessing radiological risks associated with radioactivity depends essentially on some fundamental risk indices that present quantifiable parameters of the potential health risks from radionuclides. These indices help to establish rules and regulations concerning the safe applications of studied materials. The external hazard index, H_ex_, which helps in the overall assessment of external radiation exposure is evaluated using equation (8) [61].


$$H_{ex}=\;\frac{S_{Ra}}{370}+\;\frac{S_{Th}}{259}+\frac{S_{K}}{4810}$$ (8)

Equation (9) yields the internal hazard index (H_in_) [62].


$$H_{in}=\;\frac{S_{Ra}}{185}+\;\frac{S_{Th}}{259}+\frac{S_{K}}{4810}$$ (9)

#### 2.4.5 Gamma level index (I_γ_).

The gamma level index in soil assesses the distribution of radionuclides, which is crucial for understanding potential health hazards from radioactivity. The gamma level index is determined by Eqs. (10) [63,64].


$$I_{\gamma }=\;\frac{S_{Ra}}{150}+\;\frac{S_{Th}}{100}+\frac{S_{K}}{1500}$$ (10)

## 3. Results and discussion

Twenty soil samples were collected from two industrial zones in the Chattogram City of Bangladesh: the Bayazid Industrial Area and the Kalurghat Heavy Industrial Area. The activity concentrations measured in these soil samples are presented in Table 1.

**Table 1:** Activity Concentrations of ^226^Ra, ^232^Th, and ^40^K in Soil Samples with Associated Uncertainties.

SL	Location of the collected sample	Latitude (N)	Longitude (E)	^226^Ra (Bq/kg)	^232^Th (Bq/kg)	^40^K (Bq/kg)
1	Kalurghat	22.39369°	91.86458°	18 ± 1	20 ± 2	192 ± 17
2		22.39776°	91.86596°	15 ± 1	24 ± 2	320 ± 31
3		22.39833°	91.86366°	11 ± 1	15 ± 1	250 ± 23
4		22.39646°	91.86298°	14 ± 1	23 ± 2	290 ± 27
5		22.39853°	91.86105°	8 ± 1	18 ± 1	290 ± 25
6	Bayazid	22.37225°	91.81378°	11 ± 1	19 ± 1	260 ± 25
7		22.37395°	91.81267°	15 ± 1	24 ± 2	340 ± 31
8		22.37846°	91.8132°	10 ± 1	17 ± 1	270 ± 23
9		22.3786°	91.81808°	13 ± 1	20 ± 2	280 ± 25
10		22.38039°	91.81653°	13 ± 1	22 ± 2	300 ± 27
11		22.38307°	91.81091°	12 ± 1	20 ± 2	290 ± 25
12		22.38464°	91.8107°	9 ± 1	17 ± 1	250 ± 21
13		22.38456°	91.81464°	14 ± 1	24 ± 2	310 ± 29
14	Kalurghat	22.39124°	91.88017°	14 ± 1	22 ± 2	280 ± 25
15		22.39268°	91.88073°	17 ± 2	35 ± 3	420 ± 35
16		22.38859°	91.87177°	14 ± 1	24 ± 2	320 ± 29
17		22.39036°	91.87174°	10 ± 1	23 ± 2	290 ± 27
18		22.38876°	91.87654°	14 ± 1	30 ± 3	350 ± 33
19		22.38371°	91.86932°	13 ± 1	23 ± 2	330 ± 31
20		22.38212°	91.86563°	13 ± 1	20 ± 2	280 ± 27
Average				13 ± 1	22 ± 2	296 ± 25

The measured activity concentrations of ^226^Ra, ^232^Th, and ^40^K in the soil samples collected from the Bayazid and Kalurghat Heavy Industrial Area remain below the global average values of 30, 35, and 400 Bq/kg, respectively, as reported in the literature [3]. Furthermore, the absence of ^137^Cs activity in the samples eliminates the possibility of contamination from nuclear fallout, such as the Chernobyl or Fukushima incidents. The radionuclides exhibit a distinct hierarchy of activity concentrations, with ^40^K being the highest, followed by ^232^Th and ^226^Ra. This pattern reflects the geochemical characteristics and natural prevalence of these radionuclides, with ^40^K levels being higher due to the abundance of potassium in rocks and minerals. The higher activity of ^232^Th compared to ^226^Ra aligns with the known crustal abundance of thorium, which is approximately 1.5 times that of uranium [12].

While industrial zones are often associated with elevated radioactivity [65,66] due to anthropogenic and natural factors, the absence of specific conditions in the studied locations explains the relatively low activity levels. Industries such as oil and gas, mining, or phosphate fertilizer production, which bring NORMs to the surface, are not present in the Bayazid and Kalurghat industrial areas. Similarly, activities involving the use of radioactive isotopes, such as in nuclear power plants or medical facilities, are absent in these regions. Improper disposal of industrial waste, a common contributor to elevated radioactivity, is not evident in the study area, and no waste containing radioactive byproducts, such as fly ash, has been identified. Additionally, the natural geology of the studied regions does not indicate the presence of uranium- or thorium-rich minerals that could contribute to high radioactivity levels.

The geochemical properties of the radionuclides further influence their distribution. The relatively low ^226^Ra concentrations are likely due to the limited interaction between the soil and uranium-bearing minerals. Similarly, the low ^232^Th levels suggest the non-abundance of thorium-rich minerals such as monazite in the local geology. Minor variations in radionuclide distribution across soil samples may be attributed to differences in geological and topographical characteristics within the area [4,6,51,62,67–72]. These findings indicate that the Bayazid and Kalurghat industrial zones do not exhibit the conditions typically associated with high radiological hazards, making them safer in comparison to regions with significant industrial or geological contributions to elevated radioactivity.

The following Table 2 compares the activity concentration of soil samples from industrial areas in various countries worldwide with the findings of the current study, based on previously published literature.

**Table 2:** Comparison of the range (average) of ²²⁶Ra, ²³²Th, and ⁴⁰K activity concentrations in soil from industrial areas across different countries with the findings of the present study.

Sl no.	Location of the industry	Type of Industry	Range (Average) of activity concentration (Bq/kg)				Reference
			^226^Ra	^232^Th	^40^K	^137^Cs	
01.	Port Said city, Egypt	Dumping sites	18.03- 398.66	5.28-75.7	583.12-3237.88	–	[2]
02.	Industries in Northern Al Jubail, Saudi Arabia.	Petrochemical, iron, and chemical manufacturing industries; wastewater treatment facilities, gas plants, oil refineries, and ethylene and methanol production plants.	4.09 ± 0.2 −17.5 ± 0.5 (7.64 ± 0.4)	2.23 ± 0.1 −5.81 ± 0.2 (3.76 ± 0.2)	125 ± 2.4 −256 ± 4.3	MDA-0.916 ± 0.02	[31]
03.	Islamabad’s industrial Area, Pakistan	Steel mills, chemical plants, battery manufacturers, pipe production, polymer industries, pharmaceuticals, marble processing, and diesel generator factories.	10.43 ± 7.0 39.72 ± 20 (25.96 ± 12.50)	6.72 ± 2.00 −20.19 ± 4 (15.84 ± 2.59)	343 ± 38-559 ± 62 (469.48 ± 52.38)	–	[73]
04.	Severodvinsk industrial district, NW Russia	Ship repair center, machinery manufacturing facility, and radioactive waste storage site.	1.0-23.8 (11.9)	3.8-15.7 (8.7)	85.9-375.6 (191.3)	1.4-188.6 (51.9)	[29]
05.	Sethiyathope, Tamilnadu, India	Sugar mill, chemical industries	20.28-24.72 (22.8)	37.3-43.2 (39.9)	220.9-270.3 (253.16)	–	[74]
06.	Odiel and Tinto River, southwest of Spain	Chemical facilities for producing phosphoric acid and phosphate fertilizers.	66.0 ± 3.5-225 ± 11 (119 ± 16)	19.9 ± 1.7-58.1 ± 4.0 (49.3 ± 3.0)	235 ± 14- 402 ± 24 360 ± 12	0.4 ± 0.2- 5.1 ± 0.6 (2.5 ± 0.5)	[28]
07.	Agbara Industrial Estate, Ogun State, Nigeria	Food and beverages, pharmaceutical industries	1.53- 10.17 (5.05)	3.19-17.73 (9.11)	57.88-397.51 (171.33)	–	[75]
08.	Industrial Park of northwest China	Zinc extraction, iron production, coke manufacturing, and coal-based power generation.	30.2-37.5 (33.2)	56.5-79.8 (71.8)	785.6-940.3 (866.2)	–	[30]
09.	Fashina Village, Ile-Ife, Osun State, Nigeria	Iron and Steel Smelting Area	7.28 ± 2.10-20.18 ± 6.74 (12.14 ± 4.17)	6.28 ± 2.15-41.22 ± 12.6 (23.23 ± 7.67)	92.85 ± 29.06-429.07 ± 84.63 (270.14 ± 61.79)	–	[76]
10.	Agbara Industrial Estate, Ogun State, Nigeria	Food and beverages, pharmaceutical industries	BDL- 72.878 ± 23.17 (20.01)	BDL-118.29 ± 13.30 (52.90)	472.14 ± 108.36 (177.87)	–	[77]
11.	The industrial zone of the Federal Capital Territory (FCT) in Abuja, Nigeria.	Agro-allied and household item production industries	9.2 ± 0.7- 41.1 ± 4.4 (21.57)	71.1 ± 2.2- 107.3 ± 2.2 (85.87)	223.5 ± 8.4-943.1 ± 5.3 (539.57)	–	[78]
12.	Riverbank soil from different industrial areas in the heavily populated cities of Dhaka and Chattogram, Bangladesh.	Garments, textiles, cement, brick klin	27-47 (37)]	47-68 (58)	982-1359 (1129)	–	[5]
13.	Around the Turag River, Savar, Bangladesh	Industrial effluents of the adjacent areas	32.29 ± 3.92-73.94 ± 6.51 (52.22 ± 5.48)	59.76 ± 6.78-140.22 ± 10.41 (90.65 ± 8.75)	662.53 ± 88.12-1130.53 ± 159.26 (870.45 ± 120.45)	–	[17]
14.	Shyampur, Dhaka, Bangladesh	Steel production	6 ± 1-37 ± 3 (17)	6 ± 1-45 ± 4 (23)	101 ± 9-470 ± 45 (263)	–	[1]
15.	Savar industrial area, Bangladesh	Textile mills, leather products, metal goods, electronics, paper products, chemicals, and fertilizers.	24.75 ± 7.91-39.84 ± 11.83 (30.56 ± 8.17)	45.88 ± 14.67-73.64 ± 11.89 (60.45 ± 12.21)	415.72 ± 170.17-933.54 ± 250.32 (706.00 ± 202.67)	–	[79]
16.	Karnaphuli river near the industrial zone, Chattogram, Bangladesh	Industrial waste	40.44 ± 8.69-47.85 ± 7.09 (45.32 ± 9.74)	41.44 ± 14.21-56.93 ± 19.30 (47.34 ± 16.60)	321.7 ± 96.15-424.55 ± 89.03 (389.57 ± 117.14)	–	[80]
17.	Karnaphuli river sediment, Chattogram, Bangladesh	Municipal and Industrial waste	22.28 + 6.03-132.42 + 14.55 (61.02 + 9.33)	41.52 + 3.53-106.62 + 8.42 (79.68 + 6.44)	370.07 + 32.16- 1207.40 + 76.82 (856.88 + 59.45)	–	[80]
18.	Nasirabad Industrial Area, Chattogram, Bangladesh	Steel manufacturing, metal and plastic fabrication, food processing plants, paint and chemical production facilities, oil refineries, silver recovery, woodworking, and textile factories.	8 ± 2–131 ± 18.33 (21)	10 ± 2.69 −133 ± 15.96 (40)	81 ± 22.68- 930 ± 260.40 (449)	–	[81]
19.	Bayazid Industrial Area and the Kalurghat Heavy Industry Area	Steel production, textile, chemical, leather, garments	(8 ± 1-18 ± 1) (13 ± 1)	(15 ± 1-35 ± 3) 22 ± 2	192 ± 17-420 ± 35 (296 ± 25)	–	Current study

A comparative analysis of radionuclide activity concentrations in industrial soils across various countries reveals significant variations, primarily influenced by geological characteristics, industrial activities, and waste disposal practices. The highest activity concentration of ^226^Ra was observed in Port Said, Egypt, with values between 18.03 and 398.66 Bq/kg; the authors [13] propose that the elevated radium levels are likely due to the site’s proximity to areas with heavy industrial activities, such as fertilizer production, chemical factories, and a paint manufacturing plant. Other notable locations with high ²²⁶Ra levels include Odiel and Tinto River, Spain (66.0–225 Bq/kg), where chemical plants for phosphoric acid and phosphate fertilizer production contribute to radionuclide accumulation. The highest ²³²Th activity concentration was observed in the Industrial District of Abuja, Nigeria, with values ranging from 71.1 to 107.3 Bq/kg [78]. This is likely due to the presence of natural thorium-bearing minerals in the soil, combined with industrial waste discharge from agro-allied and household item production industries. Other areas with high ²³²Th levels include the Odiel and Tinto River in Spain (19.9–58.1 Bq/kg) [28] and the Industrial Park of Northwest China (56.5–79.8 Bq/kg) [30], both of which have industries that process raw materials containing thorium-rich minerals. The highest ⁴⁰K concentration was recorded in Port Said, Egypt, with a range of 583.12 to 3237.88 Bq/kg [2]. This high value is likely due to the accumulation of potassium-rich fertilizers and industrial byproducts. The highest ¹³⁷Cs activity concentration was found in Severodvinsk, Russia, with values ranging from 1.4 to 188.6 Bq/kg [29]. This can be attributed to the presence of a radioactive waste storage facility and historical nuclear activities in the area. In contrast, other industrial regions showed minimal or undetectable levels of ¹³⁷Cs, suggesting that artificial radioactivity contamination is not widespread in non-nuclear industrial areas.

The activity concentrations of ^226^Ra, ^232^Th, and ^40^K in industrial soil across different region in Bangladesh vary significantly, depending on the type of industry and environmental factors. The highest levels were recorded in the Karnaphuli river sediment, Chattogram (22.28–132.42 Bq/kg) [80], likely due to municipal and industrial waste disposal. In comparison, the Savar Industrial Area exhibited moderate levels (24.75–39.84 Bq/kg) [79], while the current study area had lower values (8–18 Bq/kg). The Turag River industrial zone reported the highest ²³²Th concentrations in Bangladesh (59.76–140.22 Bq/kg) [17], possibly due to industrial effluents containing thorium-rich minerals. In contrast, the present study recorded lower levels (15–35 Bq/kg), indicating a relatively lesser influence of industrial thorium contamination. The highest ⁴⁰K concentration in Bangladesh was found in the Karnaphuli river sediment (370.07–1207.40 Bq/kg) [80], likely due to potassium-rich industrial waste. In contrast, the current study area showed relatively moderate values (192–420 Bq/kg). Overall, the radionuclide concentrations observed in the present study are lower than those found in contaminated regions of Bangladesh, such as the Turag River and Karnaphuli River sediments, as well as in other parts of the world.

**Fig 4:**
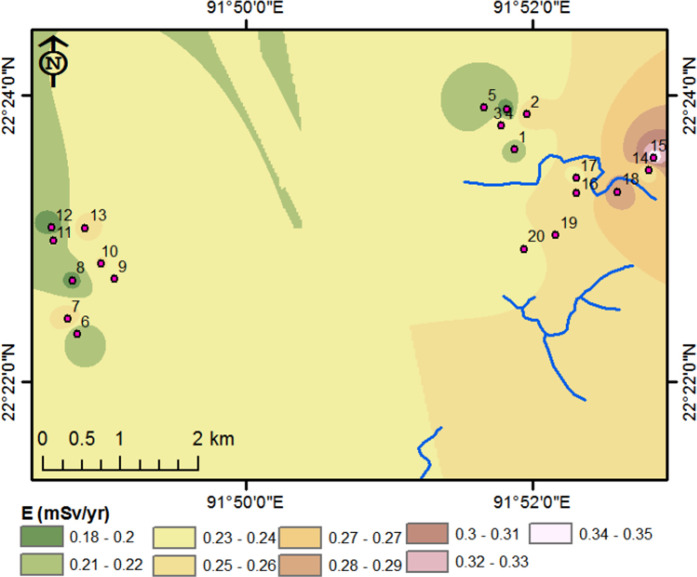
Map of the spatial distribution of total effective dose in the studied area, produced using inverse distance weighting (IDW) in ArcMap 10.2 [14,82].

The calculated values of various hazard parameters for the soil samples are provided in Table 3, with the spatial distribution of total effective dose is depicted in Fig 4.

**Table 3:** Radiological hazard parameters for soil samples in the current study.

Sample ID	Ra_eq_ mSv	D_out_ nGy/hr	D_in_ nGy/hr	H_ex_	H_in_	E_out_ mSv	E_in_ mSv	Iγ	ELCR ×10^−3^
1	61.38	29.22	35.06	0.17	0.21	0.04	0.17	0.45	0.13
2	73.96	36.12	43.34	0.20	0.24	0.04	0.21	0.55	0.16
3	51.70	25.43	30.51	0.14	0.17	0.03	0.15	0.39	0.11
4	69.22	33.73	40.48	0.19	0.22	0.04	0.20	0.52	0.15
5	56.07	27.86	33.43	0.15	0.17	0.03	0.16	0.43	0.12
6	58.19	28.51	34.21	0.16	0.19	0.04	0.17	0.44	0.13
7	75.50	36.98	44.38	0.20	0.24	0.05	0.22	0.57	0.17
8	55.10	27.19	32.63	0.15	0.18	0.03	0.16	0.42	0.12
9	63.16	30.89	37.06	0.17	0.21	0.04	0.18	0.47	0.14
10	67.56	33.08	39.69	0.18	0.22	0.04	0.19	0.51	0.15
11	62.93	30.89	37.07	0.17	0.20	0.04	0.18	0.47	0.14
12	52.56	25.90	31.08	0.14	0.17	0.03	0.15	0.40	0.12
13	72.19	35.26	42.31	0.20	0.23	0.04	0.21	0.54	0.16
14	67.02	32.64	39.17	0.18	0.22	0.04	0.19	0.50	0.15
15	99.39	48.57	58.29	0.27	0.31	0.06	0.29	0.74	0.22
16	72.96	35.69	42.83	0.20	0.23	0.04	0.21	0.55	0.16
17	65.22	32.02	38.43	0.18	0.20	0.04	0.19	0.49	0.14
18	83.85	40.96	49.15	0.23	0.26	0.05	0.24	0.63	0.18
19	71.30	35.03	42.04	0.19	0.23	0.04	0.21	0.54	0.16
20	63.16	30.89	37.06	0.17	0.21	0.04	0.18	0.47	0.14
Avg	67.12	32.84	39.41	0.18	0.22	0.04	0.19	0.50	0.15
World average (82)	370	59	84	<1	<1	0.07	0.41	<1	0.29

The comprehensive evaluation of radiation hazard parameters, as detailed in Table 3 and illustrated in Fig 4, demonstrates that the radiation hazard values associated with the soil samples are well within the safety thresholds established by several reputable international organizations [83,84]. This analysis collectively indicates that the radiation levels in the soil samples from the industrial areas of Chattogram, Bangladesh do not pose any significant immediate risk to public health or the environment. Consequently, the results provide reassurance that the soil from these areas does not require any special precautions or remediation. Given the absence of notable radiation hazards, all the examined soil samples are considered suitable for practical uses such as agricultural activities or construction purposes. This finding is especially important for promoting sustainable development in the region, as it ensures that the land can be safely utilized without compromising the well-being of the local population or the surrounding environment.

## 4. Statistical analysis

### 4.1 Descriptive statistics

Researchers typically employ both univariate and multivariate statistical techniques to comprehensively analyze the relationships between various radiological parameters when evaluating natural radiation levels in environmental matrices such as soil, sand, and water. This study performed a thorough investigation into the concentrations of radioisotopes (^226^Ra, ^232^Th, and ^40^K) and assessed the potential radiological hazards associated with them. The analysis involved a detailed examination of a standardized dataset (Table 4) using various quantitative measures namely mean, median, mode, standard deviation, kurtosis, and skewness, as well as dendrogram hierarchical cluster analysis. The close alignment between the mean and median values for each NORM indicates a robust normal distribution.

**Table 4:** Statistical overview of NORMs in Study Samples.

Parameters	^226^Ra	^232^Th	^40^K
Mean	12.90	22.00	295.60
Median	13.00	22.00	290.00
Std. Deviation	2.532	4.542	46.270
Variance	6.411	20.632	2140.884
Skewness	−.048	1.266	.514
Kurtosis	−.056	2.702	2.455
Minimum	8	15	192
Maximum	18	35	420

**Fig 5:**
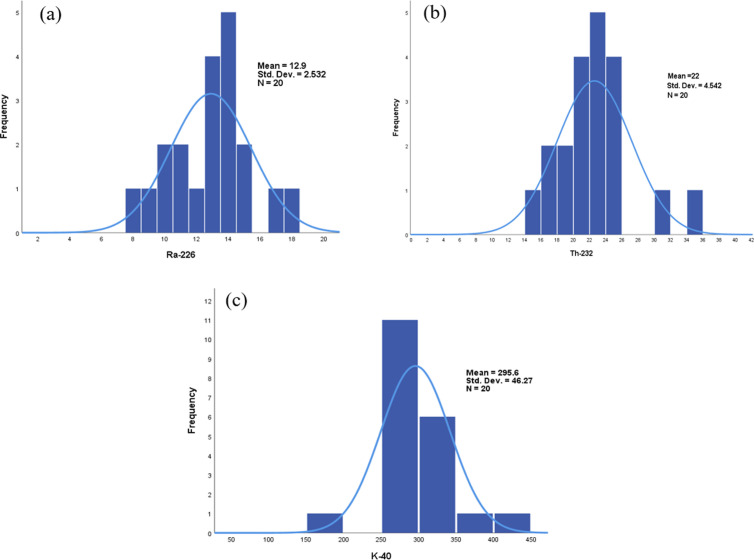
Histogram of Frequency Distribution for (a) ^226^Ra; (b) ^232^Th; (c) ^40^K.

Skewness measures the asymmetry in data, indicating deviation from a normal distribution. A distribution can exhibit either positive or negative skewness. Positive skewness signifies that the distribution leans to the right, with the mean surpassing both the mode and median. In contrast, negative skewness means the distribution tilts to the left, with the mode exceeding the mean and median. The positive skewness observed in ^232^Th and ^40^K shows that ^232^Th has the highest skewness values. Figs 5(a), 5(b), and 5(c) display the frequency distribution histograms for ^226^Ra, ^232^Th, and ^40^K respectively. These histograms support our findings regarding the distribution of these NORMs in the soils from the sampling area at Chattogram.

Kurtosis quantifies the extent of peakedness in a frequency distribution by analyzing the shape of its curve. Kurtosis is classified into three categories: A curve with a relatively sharp peak is referred to as leptokurtic, while a curve with a flat-topped peak is termed as platykurtic [85]. A normal distribution curve is known as mesokurtic. The positive kurtosis values observed for ^232^Th, and ^40^K indicate a leptokurtic distribution, characterized by a sharp peak (kurt  > 0). In contrast, the negative kurtosis values for ^226^Ra suggest a platykurtic distribution, with a flatter peak (kurt  < 0).

### 4.2 Pearson’s correlations study

**Fig 6:**
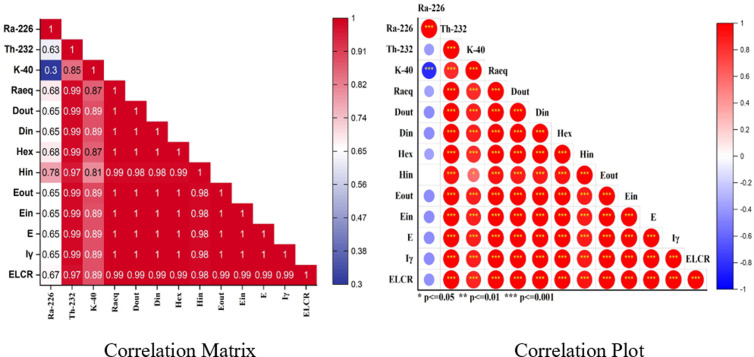
Pearson correlation between NORM and related radioactive variables.

The Pearson correlation coefficient (*r*) was employed to quantify the strength of the linear association between the NORMs and related radioactive variables. The correlation coefficients are visualized in Fig 6, where positive correlations (the correlation plot) are shown in red and negative correlations in blue, with the intensity reflecting the strength of the correlation. Significant correlations are marked based on their p-values (*p ≤ 0.05, **p ≤ 0.01, ***p ≤ 0.001). The positive correlation coefficient (*r* > 0.75, *p*  < 0.001) between ^232^Th and ^40^K reflects a strong relationship, implying that their concentrations in soil are probably derived from similar origins and factors [86]. There was a weak correlation observed between ^226^Ra and ^232^Th (0.63 < *r* < 0.75), ^40^K (0.30 < *r* < 0.75), indicating that ^226^Ra has a different source or influence in the study samples. The radionuclides ^232^Th and ^40^K showed a strong positive correlation with all radiological factors (*r* = 0.85 > 0.75), indicates that the levels of K and Th are closely related to and influence the radiological hazards. In contrast, ^226^Ra exhibited a weak correlation with radiological hazard parameters, suggesting that ^226^Ra possess minimal impact on radiological risks. Radiological hazard parameters, including hazard indices (H_in_ and H_ex_), dose rates (D_in_ and D_out_), gamma radiation index (I_γ_), effective dose (E) showed strong correlations with ^226^Ra, ^232^Th and Ra_eq_ (r > 0.9, p < 0.001) reinforcing their interdependence in assessing gamma radiation hazards.

### 4.3 Hierarchical Cluster Analysis (HCA)

**Fig 7:**
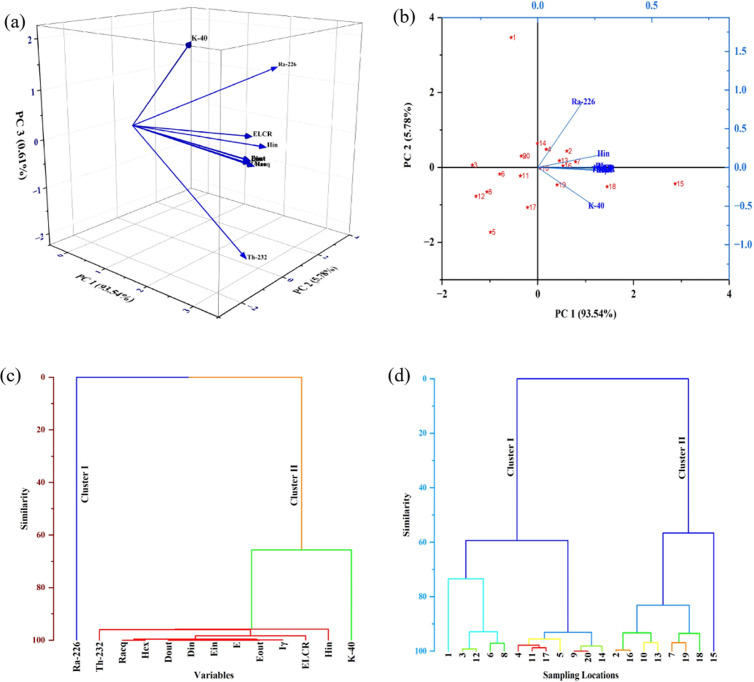
(a) 3D PCA Loading Plot of Radiological Parameters; (b) 2D PCA Biplot of Radiological Parameters and Sampling Locations; (c) HCA Dendrogram of Radiological Parameters; (d) HCA Dendrogram of Sampling Locations.

Hierarchical cluster analysis is a clustering method that organizes objects into a tree-like structure based on their similar characteristics. The goal of cluster analysis is to illustrate the hierarchical relationships among objects, ensuring that the clusters are distinctly different from each other [87]. Clusters can be created from a standardized dataset and are effectively visualized and analyzed with a dendrogram. This study employed hierarchical clustering with Ward’s method, utilizing the same variables as in the Pearson correlation analysis. Figs 7(c) and 7(d) shows two statistically significant clusters of radiological parameters and sampling locations based on Euclidean similarity. Cluster II include the ^40^K and ^232^Th radioisotopes, as well as all key radiological hazard variables, which show a strong similarity. This highlights that the increased levels of E, H_in_, H_ex_ I_γ_, D_in_, D_out_, ELCR and overall soil radioactivity are primarily due to the concentrations of ^232^Th compared to ^40^K. Cluster I consist only of ^226^Ra, suggesting that the health risks from radioactive decay are unaffected by the amount of ^226^Ra present in the sand. These findings closely align with the outcomes of the Pearson correlation analysis. Investigating these discrepancies could offer important insights into the mechanisms driving the formation and differentiation of these clusters.

### 4.4 Principal Component Analysis (PCA)

Principal Component Analysis (PCA) is a widely recognized multivariate statistical technique used to emphasize variations and identify significant patterns in a dataset, simplifying its analysis and interpretation. In this study, 3D PCA loading plot and 2D biplot was conducted with varimax rotation and employing Kaiser normalization [88]. The explained variance for PC1, PC2 and PC3 are reported in Table 5. The first principal component (PC1), explaining the majority (93.54%) of the total variance, is predominantly influenced by ^226^Ra, ^232^Th, hazard indices (H_in,_ H_ex_) and ELCR, indicating their strong interdependence and contribution to radiological hazards. while PC2 (5.78% of variance) accounts for minor variations. In contrast PC2 explains 5.78% of the variance, captures minor variations with ^40^K, exhibiting a distinct loading that indicates its weaker association with these parameters. Fig 5(a) illustrates the 3D loading plot, highlighting the contributions of each radiological parameter to the principal components (PCs), while Fig 5(b) displays the 2D biplot, mapping sampling locations alongside radiological parameters. Sampling locations cluster are grouped based on their radiological characteristics, through certain outliers (e.g., locations 1 and 15) showing unique properties likely influenced by environmental or anthropogenic factors. This analysis highlights the dominant roles of ^226^Ra, ^232^Th and related hazard parameters in contributing to radiological risks while demonstrating the variability in sampling locations, influenced by geochemical and environmental heterogeneity.

**Table 5:** Rotated loading values of radioactive variables from PCs BIPLOT ANALYSIS.

Radiological Parameters	PC1	PC2	PC3
^226^Ra	1.95123	3.46584	1.22019
^232^Th	2.82427	−0.15436	−2.09446
^40^K	2.52184	−2.09818	2.21412
Ra_eq_	2.87021	0.02643	−0.23361
D_out_	2.86893	−0.1671	−0.10143
D_in_	2.86893	−0.1671	−0.10143
H_ex_	2.87021	0.02867	−0.23189
H_in_	2.84033	0.69338	0.03576
E_out_	2.86879	−0.17539	−0.08776
E_in_	2.86894	−0.16662	−0.09985
E	2.86892	−0.16813	−0.09776
I_γ_	2.86729	−0.17982	−0.15085
ELCR	2.85644	−0.07879	0.35656
Cumulative Variance (%)	93.54%	5.78%	0.61%

### 4.5 Analysis of variance for zonal disparities

**Fig 8:**
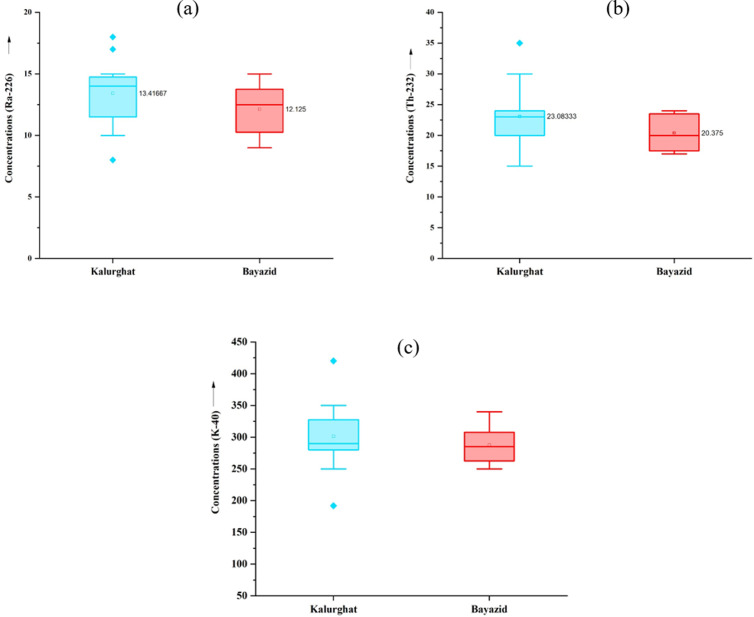
Box plots showing the variation of (a) ^226^Ra; (b) ^232^Th; (c) ^40^K with sampling locations.

In ANOVA (Analysis of Variance), a significance level of 0.05 meaning there is a chance of incorrectly rejecting the null hypothesis. The F-value indicates the probability of differences among group means, while the F-critical value, based on the F-distribution, acts as the cutoff point. When the F-value surpasses the F-critical value, it implies the presence of significant group differences. In this study, a one-way ANOVA was conducted (Table 6) to examine the zonal disparities in radionuclide levels across the industrial sites (Kalurghat and Bayazid) in Chattogram. In ANOVA, box plots are commonly used to visually compare the distribution of data across different groups. The horizontal line inside each box represents the median of the dataset displayed in Figs 8(a), 8(b), 8(c). Differences in the position of the boxes, median lines, and spread suggest variations in concentration levels between the two industrial sites. For the radionuclides ^226^Ra, ^232^Th and ^40^K, the F-values were found to be less than the critical *F*-value, and the *P*-values exceeded the significance level of 0.05. This indicates that the null hypothesis could not be rejected in any of the cases, suggesting that there is no statistically significant variation among the groups for any of the radionuclides.

**Table 6:** Overall ANOVA for radionuclide levels across the Kalurghat and Bayazid industrial sites.

Radionuclides	F Value	F_Critical_ Value	P value	Observations
^226^Ra	1.266	4.41	0.275	Failing to reject the null hypothesis
^232^Th	1.776	4.41	0.199	Failing to reject the null hypothesis
^40^K	0.395	4.41	0.54	Failing to reject the null hypothesis

## 5. Conclusion

This study presents a comprehensive assessment of NORM in twenty soil samples collected from two major industrial zones in Chattogram, Bangladesh, using a high-resolution high-purity germanium (HPGe) detector. The measured average activity concentrations of radionuclides—namely ^226^Ra (13 ± 1 Bq/kg), ^232^Th (22 ± 2 Bq/kg), and ^40^K (296 ± 25 Bq/kg)-were all found to be below the worldwide average values reported by UNSCEAR. Furthermore, no traces of artificial radioactivity, such as ^137^Cs, were detected, affirming the natural origin of the observed radionuclides and indicating an absence of nuclear contamination in the sampled areas. The analysis of radiological hazard indices-including radium equivalent activity, hazard indices, absorbed gamma dose rate, and annual effective dose-confirmed that all values were within internationally recommended safety limits. This suggests that, under current conditions, the investigated areas do not pose any significant radiological health risk to the public or to the surrounding environment. Beyond mere quantification, the study applied rigorous statistical and multivariate analyses-including Pearson’s correlation, hierarchical cluster analysis (HCA), and principal component analysis (PCA)-to explore the interrelationships and potential sources of these radionuclides. These methods collectively revealed that ^40^K and ^232^Th are the primary contributors to radiological hazards in the area. This finding is substantiated by their strong positive correlations with hazard indices, their tendency to cluster together, and their significant loadings on the principal components, suggesting similar geochemical behaviors or shared natural sources. Importantly, this is the first study of its kind conducted in the industrial regions of Chattogram. As such, it offers crucial baseline data that will be invaluable for future environmental monitoring, especially in light of Bangladesh’s growing industrialization and the upcoming commissioning of the Rooppur Nuclear Power Plant in 2025. Establishing this reference point is essential for detecting any future changes in environmental radioactivity that may arise from anthropogenic or natural factors. This research underscores the necessity of expanding such monitoring efforts to include other industrial areas, especially those adjacent to residential neighborhoods. Continuous surveillance is vital to preemptively identify any radiological threats and to protect public health as industrial activities intensify. Future studies should also consider seasonal and depth-wise variations in radionuclide distribution to develop a more dynamic and long-term understanding of environmental radioactivity inventory in Bangladesh.

## References

[1] Siraz MMM, Alam MS, Al Mahmud J, Rashid MdB, Hossain Z, AbdElrahim E, et al. Assessing radioactivity in soil in the vicinity of steel production industries: a pioneering investigation in Bangladesh. Int J Environ Analytical Chem. 2023;105(7):1469–88. doi: 10.1080/03067319.2023.2293902

[2] Attia TE, Shendi EH, Shehata MA. Assessment of natural and artificial radioactivity levels and radiation hazards and their relation to heavy metals in the industrial area of Port Said city, Egypt. Environ Sci Pollut Res Int. 2015;22(4):3082–97. doi: 10.1007/s11356-014-3453-z 25233912

[3] UNSCEAR. Sources and Effects of Ionizing Radiation, United Nations Scientific Committee on the Effects of Atomic Radiation, Annex B. 2000. 10.1088/0952-4746/21/1/609 11281539

[4] Al Mahmud J, Siraz MMM, Alam MS, Das SC, Bradley DA, Khandaker MU, et al. A study into the long-overlooked carcinogenic radon in bottled water and deep well water in Dhaka, Bangladesh. Int J Environ Analytical Chem. 2023;104(18):7161–73. doi: 10.1080/03067319.2022.2163895

[5] Shelley A, Ovi MH, Alam MS. Assessment of radioactivity level and associated radiological hazard in riverbed samples within industrial areas. Isotopes Environ Health Stud. 2024;60(2):213–25. doi: 10.1080/10256016.2024.2317391 38372986

[6] Alam MS, Siraz MMM, A M J, Das SC, Bradley DA, Khandaker MU, et al. A study on measuring the 222Rn in the Buriganga River and tap water of the megacity Dhaka. PLoS One. 2023;18(5):e0286267. doi: 10.1371/journal.pone.0286267 37220107 PMC10204947

[7] Awad HA, Abu El-Leil I, Nastavkin AV, Tolba A, Kamel M, El-Wardany RM, et al. Corrigendum to “Statistical analysis on the radiological assessment and geochemical studies of granite rocks in the north of Um Taghir area, Eastern Desert, Egypt”. Open Chem. 2022;20(1):330–330. doi: 10.1515/chem-2022-0552

[8] Zakaly HMH, Awad HA, Abbasi A, Almousa N, Elsaman R, Abd El-Salam LM, et al. Radioactive and mineralogical assessment of mediterranean black sands: a systematic analysis and health risk evaluation. J Radioanal Nucl Chem. 2024;333(4):1937–47.

[9] UNSCEAR. Sources and effects of ionizing radiation united Nations Scientific Committee on the Effects of Atomic Radiation. 2010:I;156 p. 10.1088/0952-4746/21/1/609 11281539

[10] Seif RA, Ene A, Zakaly HMH, Sallam AM, Taalab SA, Fnais MS, et al. Distribution of heavy metals along the Mediterranean shoreline from Baltim to El-Burullus (Egypt): consequences for possible contamination. Miner. 2024;14(9):931.

[11] Timmermans CWM, van der Steen J. Environmental and occupational impacts of natural radioactivity from some non-nuclear industries in the Netherlands. J Environ Radioactivity. 1996;32(1–2):97–104. doi: 10.1016/0265-931x(95)00082-l

[12] Yasmin S, Barua BS, Uddin Khandaker M, Kamal M, Abdur Rashid Md, Abdul Sani SF, et al. The presence of radioactive materials in soil, sand and sediment samples of Potenga sea beach area, Chittagong, Bangladesh: Geological characteristics and environmental implication. Results Phys. 2018;8:1268–74. doi: 10.1016/j.rinp.2018.02.013

[13] Siraz MMM, Reza A, Khan M, Alam MS, Al Mahmud J, Rashid MB. Pioneering study of radioactivity in soil near the Payra 1320 MW thermal power plant, the largest coal-fired thermal power plant in Bangladesh. Int J Environ Anal Chem. 2024;:1–19.

[14] Habib MA, Khan R, Phoungthong K. Evaluation of environmental radioactivity in soils around a coal burning power plant and a coal mining area in Barapukuria, Bangladesh: Radiological risks assessment. Chem Geol. 2022;600(December 2021).

[15] Habib MdA, Basuki T, Miyashita S, Bekelesi W, Nakashima S, Phoungthong K, et al. Distribution of naturally occurring radionuclides in soil around a coal-based power plant and their potential radiological risk assessment. Radiochimica Acta. 2018;107(3):243–59. doi: 10.1515/ract-2018-3044

[16] Rahman L, Fedous J, Begum A, Haque MM. Natural radioactivity levels and radiological health implications around the mining area of Bangladesh. Int J Innov Stud Sci Eng Technol. 2019;4863:10–5.

[17] Jahan I, Ali ML, Haydar MA, Ali MI, Paul D, Islam SMA. Distribution of natural and probable artificial radioactivity in the sediment and water samples collected from low-lying areas of Savar industrial zone, Bangladesh. J Nucl Part Phys. 2016;6(2):25–34.

[18] Faisal R, Majumder RK, Paul DC, Faisal BMR, Majumder RK, Uddin MJ. Assessment of heavy metals pollution and natural radioactivity in topsoil of Savar industrial area, Bangladesh. Int J Environ Sci. 2015;5(5):964–79.

[19] Bem H, Wieczorkowski P, Budzanowski M. Evaluation of technologically enhanced natural radiation near the coal-fired power plants in the Lodz region of Poland. J Environ Radioact. 2002;61(2):191–201. doi: 10.1016/s0265-931x(01)00126-6 12066980

[20] Flues M, Moraes V, Mazzilli BP. The influence of a coal-fired power plant operation on radionuclide concentrations in soil. J Environ Radioact. 2002;63(3):285–94. doi: 10.1016/s0265-931x(02)00035-8 12440517

[21] Papp Z, Dezso Z, Daróczy S. Significant radioactive contamination of soil around a coal-fired thermal power plant. J Environ Radioact. 2002;59(2):191–205. doi: 10.1016/s0265-931x(01)00071-6 11900206

[22] Gür F, Yaprak G. Natural radionuclide emission from coal-fired power plants in the southwestern of Turkey and the population exposure to external radiation in their vicinity. J Environ Sci Health A Tox Hazard Subst Environ Eng. 2010;45(14):1900–8. doi: 10.1080/10934529.2010.520608 20981605

[23] Lu X, Zhao C, Chen C, Liu W. Radioactivity level of soil around Baqiao coal-fired power plant in China. Radiat Phys Chem. 2012;81(12).

[24] Gören E, Turhan Ş, Kurnaz A, Garad AMK, Duran C, Uğur FA, et al. Environmental evaluation of natural radioactivity in soil near a lignite-burning power plant in Turkey. Appl Radiat Isot. 2017;129:13–8. doi: 10.1016/j.apradiso.2017.07.059 28797910

[25] Ćujić M, Dragović S, Đorđević M, Dragović R, Gajić B, Miljanić Š. Radionuclides in the soil around the largest coal-fired power plant in Serbia: radiological hazard, relationship with soil characteristics and spatial distribution. Environ Sci Pollut Res Int. 2015;22(13):10317–30. doi: 10.1007/s11356-014-3888-2 25716901

[26] Papastefanou C. Escaping radioactivity from coal-fired power plants (CPPs) due to coal burning and the associated hazards: a review. J Environ Radioact. 2010;101(3):191–200. doi: 10.1016/j.jenvrad.2009.11.006 20005612

[27] Lu X, Liu W, Zhao C, Chen C. Environmental assessment of heavy metal and natural radioactivity in soil around a coal-fired power plant in China. J Radioanal Nucl Chem. 2013;295(3):1845–54.

[28] Bolívar JP, García-Tenorio R, Mas JL, Vaca F. Radioactive impact in sediments from an estuarine system affected by industrial wastes releases. Environ Int. 2002;27(8):639–45. doi: 10.1016/s0160-4120(01)00123-4 11934113

[29] Yakovlev EYu, Zykova EN, Zykov SB, Malkov AV, Bazhenov AV. Heavy metals and radionuclides distribution and environmental risk assessment in soils of the Severodvinsk industrial district, NW Russia. Environ Earth Sci. 2020;79(10). doi: 10.1007/s12665-020-08967-8

[30] Lu X, Pan H, Ren C, Yang L. Natural radioactivity in reservoir sediment near an industrial park of northwest China. J Radiol Prot. 2016;36(2):N26–33. doi: 10.1088/0952-4746/36/2/N26 27122204

[31] Alshahri F. Natural and anthropogenic radionuclides in urban soil around non-nuclear industries (Northern Al Jubail), Saudi Arabia: assessment of health risk. Environ Sci Pollut Res Int. 2019;26(36):36226–35. doi: 10.1007/s11356-019-06647-0 31713138

[32] Rashed-Nizam QM, Rahman MM, Kamal M, Chowdhury MI. Assessment of radionuclides in the soil of residential areas of the Chittagong metropolitan city, Bangladesh and evaluation of associated radiological risk. J Radiat Res. 2015;56(1):22–9. doi: 10.1093/jrr/rru073 25237039 PMC4572591

[33] Siddique MAB, Khan R, Islam ARMT, Alam MK, Islam MS, Hossain MS. Quality assessment of freshwaters from a coastal city of southern Bangladesh: irrigation feasibility and preliminary health risks appraisal. Environ Nanotechnology, Monit Manag. 2021;16:100524.

[34] Khan MR. Plate tectonics and Bangladesh. J Asiat Soc Bangladesh Sci. 2002;28(2):39–62.

[35] Rashid MB, Habib MA, Mahmud A, Ahsan MK, Khasru MH, Hossain MA. Tectonic setting, provenance, depositional, and paleo-climatic conditions of the late quaternary subcrop sediments of the southeastern coastal region of the Bengal basin. Heliyon. 2023;9(1):e12998. doi: 10.1016/j.heliyon.2023.e12998 PMC9871222 36704270

[36] Siraz MMM, A M J, Alam MS, Rashid MB, Hossain Z, Khandaker MU, et al. Measurement of radioactivity in soils of Karamjal and Harbaria mangrove forest of Sundarbans for establishment of radiological database. PLoS One. 2023;18(10):e0289113. doi: 10.1371/journal.pone.0289113 37856554 PMC10586596

[37] Siraz MMM, Rakib MDA, Alam MS, Al Mahmud J, Rashid MB, Khandaker MU, et al. Assessment of radionuclides from coal-fired brick kilns on the outskirts of Dhaka city and the consequent hazards on human health and the environment. Nucl Eng Technol. 2023.

[38] Siraz MMM, Al Mahmud J, Alam MS, Rashid MB, Hossain Z, Khandaker MU. Risk assessment of naturally occurring radioactivity in soil adjacent to a coal-fired brick kiln. Radiat Phys Chem. 2023;209:110985.

[39] Siraz MMM, Fahim MR, Khan ZH, Alam MS, Mahmud A, Rashid MB. Assessment of soil radioactivity and associated health risks in the Haripur gas field, Bangladesh. Isotopes Environ Health Stud. 2025;6016:1–21. 10.1080/10256016.2025.2501051 40391472

[40] Khandaker MU, Haba H, Kanaya J, Otuka N. Excitation functions of (d,x) nuclear reactions on natural titanium up to 24MeV. Nuclear Instruments and Methods in Physics Research Section B: Beam Interactions with Materials and Atoms. 2013;296:14–21. doi: 10.1016/j.nimb.2012.12.003

[41] Yeasmin S, Karmaker S, Rahman AM, Siraz MMM, Sultana MS. Measurement of radioactivity in soil and vegetable samples in the northern area of Madhupur Upzila at Tangail District in Bangladesh and assessment of associated radiological. Bangladesh J Phys. 2014;16(March 2017):49–58.

[42] Aktar MN, Das SK, Yeasmin S, Siraz MM, Rahman AM. Measurement of radioactivity and assessment of radiological hazard of tea samples collected from local market In Bangladesh. J Bangladesh Acad Sci. 2018;42(2):171–6. doi: 10.3329/jbas.v42i2.40049

[43] Sultana N, Siraz MMM, Pervin S, Kabir MF, Hassan N, Banik S, et al. Real-time environmental gamma dose monitoring around dhaka university real-time environmental gamma dose monitoring around dhaka university campus and estimation of excess lifetime cancer risk on public health. J Radiat Nucl Appl. 2023;8(1):9–16.

[44] Siraz MMM, Roy D, Dewan MJ, Alam MS, A M J, Rashid MB, et al. Vertical distributions of radionuclides along the tourist-attractive Marayon Tong Hill in the Bandarban district of Bangladesh. Environ Monit Assess. 2023;195(3):382. doi: 10.1007/s10661-023-10921-7 36759352

[45] Hameed PS, Pillai GS, Mathiyarasu R. A study on the impact of phosphate fertilizers on the radioactivity profile of cultivated soils in Srirangam (Tamil Nadu, India). J Radiat Res Appl Sci. 2014;7(4):463–71.

[46] Siraz MMM, Das SK, Mondol MS, Alam MS, Al Mahmud J, Rashid MB, et al. Evaluation of transfer factors of 226Ra, 232Th, and 40K radionuclides from soil to grass and mango in the northern region of Bangladesh. Environ Monit Assess. 2023;195(5):579. doi: 10.1007/s10661-023-11223-8 37067680

[47] Asaduzzaman K, Mannan F, Khandaker MU, Farook MS, Elkezza A, Amin YBM, et al. Assessment of natural radioactivity levels and potential radiological risks of common building materials used in Bangladeshi dwellings. PLoS One. 2015;10(10):e0140667. doi: 10.1371/journal.pone.0140667 26473957 PMC4608819

[48] Khandaker MU, Heffny N, Adillah B, Amin YM, Bradley DA. Elevated concentration of radioactive potassium in edible algae cultivated in Malaysian seas and estimation of ingestion dose to humans. Algal Res. 2019;38.

[49] Siraz MMM, Kamal MH, Khan ZH, Alam MS, Al Mahmud J, Rashid MB. Radionuclide transfer in tea cultivation: assessing radiological risks in the largest and first established tea garden in Bangladesh. Soil Sediment Contam An Int J. 2024;00(00):1–19.

[50] Mahmud A, Siraz MMM, Trishna JM, Alam MS, Rashid MB, Khandaker MU, et al. Radiological impact of ship-breaking operations and container depot explosions on Sitakunda coast, Chattogram, Bangladesh: implications for public health. Int J Environ Anal Chem. 2025;00(00):1–26.

[51] Siraz MMM, Dewan MJ, Chowdhury MIA, Al Mahmud J, Alam MS, Rashid MB, et al. Radioactivity in soil and coal samples collected from the vicinity of the coal-fired thermal power plant and evaluation of the associated hazard parameters. Int J Environ Analytical Chem. 2023;104(19):8185–202. doi: 10.1080/03067319.2023.2196021

[52] Al Mahmud J, Siraz MMM, Alam MS, Dewan MJ, Rashid MB, Khandaker MU, et al. A pioneering study of the radiological mapping in the world’s largest mangrove forest (the Sundarbans) and implications for the public and environment. Mar Pollut Bull. 2024;202:116349. doi: 10.1016/j.marpolbul.2024.116349 38604081

[53] Siraz MMM, Al Mahmud J, Alam MS, Rashid MB, Hossain Z, Osman H, et al. Assessment of radioactivity level and associated radiological hazard in fertilizer from Dhaka. Environ Monit Assess]. 2024;196(2):1–13. https://www.tandfonline.com/doi/full/10.1080/10256016.2024.2317391 10.1007/s10661-024-12328-4 38263472

[54] International Commission on Radiological Protection. Annals of the ICRP. Vol. 37, ICRP Publication 103. 2007. 10.1016/j.icrp.2007.10.003 18082557

[55] Khan R, Begum A, Hoque A, Siraz MMM. Occupational dose from external ionizing radiation in interventional cardiology. Bangladesh J Phys. 2013;14:17–22.

[56] Siraz M, Begum A, Khan R, Hoque A, Begum A. Measurement of effective dose to patient during interventional cardiac procedure. J Asiat Soc Bangladesh, Sci. 2014;40(1):1–7. doi: 10.3329/jasbs.v40i1.31728

[57] Rahman MS, Begum A, Hoque A, Khan RK, Siraz MMM. Assessment of whole-body occupational radiation exposures in nuclear medicine practices of Bangladesh during 2010-2014. Iran J Nucl Med. 2016;24(1):51–8.

[58] Rahman MS, Begum A, Khan MdRK, Hoque MdA, Siraz MMM. Occupational exposure to ionizing radiation in interventional cardiology practices in Bangladesh during 2010-2014. Malays J Med Biol Res. 2016;3(2):63–8. doi: 10.18034/mjmbr.v3i2.407

[59] Rahman MS, Begum A, Hoque A, Khan RK, Siraz MMM. Assessment of whole-body occupational radiation exposure in industrial radiography practices in Bangladesh during 2010-2014. Brazilian J Radiat Sci. 2016;4(2).

[60] Hossain S, Siraz MMM, Hossain MZ, Yeasmin S. Ionizing radiation exposure at Interventional Cardiology practices in Bangladesh. ACS Chem Health Saf. 2024.

[61] Siraz MMM, Kamal MH, Khan ZH, Alam MS, Al Mahmud J, Rashid MB, et al. Evaluation of radioactivity in soil and rock samples from an undiscovered sea beach in the southeastern coastline of Bangladesh and associated health risk. Environ Monit Assess. 2023;195(9):1028. doi: 10.1007/s10661-023-11636-5 37558890

[62] Siraz MMM, Al Mahmud J, Alam MS, Rashid MdB, Hossain Z, Joydhar A, et al. Baseline radioactivity in the five candidate sites for the second nuclear power plant in Bangladesh and concomitant hazards assessment. Int J Environ Analytical Chem. 2023;104(20):8639–54. doi: 10.1080/03067319.2023.2207470

[63] Siraz MMM, Haque T, Choudhury TR, Alam MS, Mahmud A, Rashid MB. Evaluating the radioactivity and health risks in oil and gas production areas: insights from Titas Gas Field, Bangladesh. Int J Environ Anal Chem. 2024; 1–22.

[64] Yeasmin S, Das SK, Siraz MMM, Rahman AFMM, Rahman MS. Radiometric hazard assessment of soil and water samples adjacent to Bangladesh’s first nuclear power plant before commissioning: Insights into human health and environmental radiological dynamics. Heliyon. 2024;10(20):e39516. doi: 10.1016/j.heliyon.2024.e39516 39469689 PMC11513540

[65] Khandaker MU, Mahmud A, Siraz MMM, Alam MS, Trishna JM, Rashid MB. Identification of elevated level background radiation areas, exposure scenarios and implications for public health and environmental safety in Malaysia: A comprehensive study. Radiat Phys Chem. 2025;235(February):112851.

[66] Orthi KH, Sultana T, Ferdous J, Siraz MMM, Hannan A. Analysis of radionuclides and radiation hazards in soil, sediment, water, and rock in Sylhet, Bangladesh. Radiat Prot Dosimetry. 2025;201(5):386–97. doi: 10.1093/rpd/ncaf027 40151003

[67] Dos Santos Júnior JA, Dos Santos Amaral R, do Nascimento Santos JM, da Silva ANC, Rojas LAV, Milan MO, et al. radioactive disequilibrium and dynamic of natural radionuclides in soils in the state of Pernambuco-Brazil. Radiat Prot Dosimetry. 2018;182(4):448–58. doi: 10.1093/rpd/ncy101 29912424

[68] Tanha M, Riebe B, Ikeda-Ohno A, Schulze M, Khalid FR, Storai A. Environmental radioactivity studies in Kabul and northern Afghanistan. J Radioanal Nucl Chem. 2018;318(3):2425–33.

[69] Siraz MMMM, Alam MS, Jubair AM, Das SC, Ferdous J, Hossain Z. The presence of carcinogenic radon in the Padma River water, adjacent to the Rooppur Nuclear Power Plant. Nuclear Eng Technol. 2023;55(8):3046–53.

[70] Jibiri NN, Ugbechie A, Sowunmi AA, Akomolafe IR. Radionuclide contents in sediment and seafood from Makoko Lagoon, Lagos State, Nigeria. Mar Pollut Bull. 2023;192:114992. doi: 10.1016/j.marpolbul.2023.114992 37182242

[71] Şahin Bal S, Kurşat M, Kuluöztürk MF, Karatepe Çelik Ş, Yılmaz E. Soil to plant transfer of 226Ra, 232Th and 137Cs to some medicinal and aromatic plants growing in Bitlis (Turkey). J Environ Radioact. 2023;257:107089. doi: 10.1016/j.jenvrad.2022.107089 36538843

[72] Siraz MMM, Kamal MH, Khan ZH, Alam MS, Al Mahmud J, Rashid MB, et al. Evaluation of radioactivity in soil and rock samples from an undiscovered sea beach in the southeastern coastline of Bangladesh and associated health risk. Environ Monit Assess. 2023;195(9):1028. doi: 10.1007/s10661-023-11636-5 37558890

[73] Anjum M, Siddique N, Younis H, Faiz Y, Shafique MA, Mahnoor A, et al. Heavy metals and radionuclides in Islamabad’s industrial area: A comprehensive analysis of soil and water pollution, source apportionment and health effects using statistical and geospatial tools. J Trace Elements and Minerals. 2024;8:100127. doi: 10.1016/j.jtemin.2024.100127

[74] Senthilkumar RD, Narayanaswamy R. Assessment of radiological hazards in the industrial effluent disposed soil with statistical analyses. J Radiation Res Appl Sci. 2016;9(4):449–56. doi: 10.1016/j.jrras.2016.07.002

[75] Bodunrin JO, Ajayi OS. Natural radioactivity measurements to determine the radiation hazards from surface soil and effluents in Agbara industrial estate, Ogun State, Nigeria. Int J Innov Res Adv Stud. 2017;4(11).

[76] Oluyide S, Tchokossa P, Orosun M, Akinyose F, Louis H, Ige S. Natural radioactivity and radiological impact assessment of soil, food and water around iron and steel smelting area in Fashina village, Ile-Ife, Osun State, Nigeria. J Appl Sci Environ Manag. 2019;23(1):135.

[77] Environmental radioactivity monitoring and radiological impact assessment of Agbara industrial area, Ogun State, Nigeria. 2024.

[78] Umar A. Baseline measurement of natural radioactivity in soil, vegetation and water in the industrial district of the federal capital territory (FCT) Abuja, Nigeria. Br J Appl Sci Technol. 2012;2(3):266–74.

[79] Faisal BMR, Majumder RK, Uddin MJ, Deeba F, Paul D, Haydar MA. Assessment of heavy metals pollution and natural radioactivity in topsoil of Savar industrial area, Bangladesh. Int J Environ Sci. 2015;5(5):964–79.

[80] Alam MK, Chakraborty SR, Rahman AKMR, Deb AK, Kamal M, Chowdhury MI, et al. Measurement of physiochemical parameters and determination of the level of radiological threat to the population associated with the Karnaphuli River sediment containing municipal and industrial wastes of Chittagong city in Bangladesh. Radiat Prot Dosimetry. 2013;153(3):316–27. doi: 10.1093/rpd/ncs117 22807494

[81] Mojumder MDA, Ahmed M, Kamal M, Hossen MB, Rashid MdA. The activity concentrations, radiation contamination, and hazards from wastes and soil samples in nasirabad industrial area, Chattogram, Bangladesh. JEP. 2020;11(10):767–78. doi: 10.4236/jep.2020.1110047

[82] Khan R, Haydar MA, Saha S, Karim MM, Habib MA, Rashid MB. Spatial distribution and radiological risk quantification of natural radioisotopes in the St. Martin’s Island, Bangladesh. Environ Sci Eng. 2022;:369–88.

[83] UNSCEAR. Sources and effects of ionizing radiation report to the General Assembly with scientific annexes, annex-B. New York. 2000.

[84] NEA-OECD. Exposure to Radiation from Natural Radioactivity in Building Materials. 1979.

[85] El-shanshoury I, Arafat AA. Statistical analysis of natural radioactivity measurements for the soil of Marsa Alam-Shalateen Red-Sea coast area, Egypt. Int J Adv Sci Tech Res. 2018;1(8).

[86] Chandrasekaran A, Ravisankar R, Harikrishnan N, Satapathy KK, Prasad MVR, Kanagasabapathy KV. Multivariate statistical analysis of heavy metal concentration in soils of Yelagiri Hills, Tamilnadu, India--spectroscopical approach. Spectrochim Acta A Mol Biomol Spectrosc. 2015;137:589–600. doi: 10.1016/j.saa.2014.08.093 25240831

[87] Ravisankar R, Chandramohan J, Chandrasekaran A, Prince Prakash Jebakumar J, Vijayalakshmi I, Vijayagopal P. Assessments of radioactivity concentration of natural radionuclides and radiological hazard indices in sediment samples from the East coast of Tamilnadu, India with statistical approach. Mar Pollut Bull. 2015;97(1–2):419–30. 26036177 10.1016/j.marpolbul.2015.05.058

[88] Raghu Y, Chandrasekaran A, Ravisankar R. Statistical analysis of natural radioactivity data of clay samples in Tiruvannamalai, Tamilnadu, India. Acta Ecologica Sinica. 2020;40(3):254–61. doi: 10.1016/j.chnaes.2019.12.006

